# CRISPR-Cas9-Based Discovery of the Verrucosidin Biosynthesis Gene Cluster in *Penicillium polonicum*

**DOI:** 10.3389/fmicb.2021.660871

**Published:** 2021-05-21

**Authors:** Silvia Valente, Edoardo Piombo, Volker Schroeckh, Giovanna Roberta Meloni, Thorsten Heinekamp, Axel A. Brakhage, Davide Spadaro

**Affiliations:** ^1^AGROINNOVA – Centre of Competence for the Innovation in the Agro-Environmental Sector, Grugliasco, Italy; ^2^Department of Agricultural, Forest and Food Sciences, Università degli Studi di Torino, Grugliasco, Italy; ^3^Department of Molecular and Applied Microbiology, Leibniz Institute for Natural Product Research and Infection Biology – Hans Knöll Institute, Jena, Germany; ^4^Department of Microbiology and Molecular Biology, Institute for Microbiology, Friedrich Schiller University, Jena, Germany

**Keywords:** secondary metabolites, *Penicillium*, mycotoxins, CRISPR-Cas, alpha-pyrone polyketides

## Abstract

*Penicillium polonicum*, commonly found on food matrices, is a mycotoxigenic species able to produce a neurotoxin called verrucosidin. This methylated α-pyrone polyketide inhibits oxidative phosphorylation in mitochondria and thereby causes neurological diseases. Despite the importance of verrucosidin as a toxin, its biosynthetic genes have not been characterized yet. By similarity analysis with the polyketide synthase (PKS) genes for the α-pyrones aurovertin (AurA) and citreoviridin (CtvA), 16 PKS genes for putative α-pyrones were identified in the *P. polonicum* genome. A single PKS gene, *verA*, was found to be transcribed under verrucosidin-producing growth conditions. The annotated functions of the genes neighboring *verA* correspond to those required for verrucosidin biosynthesis. To prove the involvement of *verA* in verrucosidin biosynthesis, the clustered regularly interspaced short palindrome repeats (CRISPR) technology was applied to *P. polonicum*. *In vitro* reconstituted CRISPR-Cas9 was used to induce targeted gene deletions in *P. polonicum*. This approach allowed identifying and characterizing the verrucosidin biosynthetic gene cluster. *VerA* deletion mutants were no longer able to produce verrucosidin, whereas they were displaying morphological characteristics comparable with the wild-type strain. The available CRISPR-Cas9 technology allows characterizing the biosynthetic potential of *P. polonicum* as a valuable source of novel compounds.

## Introduction

*Penicillium polonicum* is a ubiquitous fungus, found as a contaminant of food matrices, such as meat (Sunesen and Stahnke, 2003; Wigmann et al., 2018), nuts (Prencipe et al., 2018), and fresh fruit, like grape berries and strawberries (Jensen et al., 2013; Santini et al., 2014). It is also reported as a postharvest pathogen on apple (Ouhibi et al., 2018), pear (Scholtz and Korsten, 2016), onion (Duduk et al., 2014), chestnut (Prencipe et al., 2018), and cactus pear (Faedda et al., 2015). The fungus can grow as saprophyte in diverse environments (Sonjak et al., 2006). *P. polonicum* is able to produce a variety of secondary metabolites (SMs), including mycotoxins, which exhibit toxic effects on animals and humans. Characteristic compounds produced by *P. polonicum* are penicillic acid, aspterric acid, verrucofortine, cyclopenins, nephrotoxic glycopeptides, and verrucosidin (Frisvad et al., 2004), a neurotoxic compound initially isolated from *Penicillium verrucosum* var. *cyclopium*, and later from other *Penicillium* species, such as *P*enicillium *expansum* (Burka et al., 1983; Frisvad et al., 2004; Kim et al., 2016).

*Penicillium polonicum* strain X6 was previously identified, by adopting a polyphasic approach based on molecular, morphological, and chemical characterization (Prencipe et al., 2018). A multilocus phylogenetic analysis was performed using internal transcribed spacer (ITS) region, calmodulin, and β-tubulin partial genes. Morphological analysis was conducted on three different media: malt extract agar (MEA), czapek yeast agar (CYA), and yeast extract sucrose (YES). Finally, the SMs produced *in vivo* were analyzed, confirming the ability of this strain to produce verrucosidin.

From the chemical point of view, verrucosidin is a highly reduced polyketide, composed of a methylated α-pyrone, a polyene linker, and an epoxidated tetrahydrofuran ring. Many polyketides share the α-pyrone structure, such as citreoviridin and aurovertins, and have been extensively studied because of their ability to inhibit mitochondrial oxidative phosphorylation, resulting in cytotoxicity and potential antitumor activity (Li et al., 2018). Verrucosidin acts on the central nervous system, causing neurological diseases, first diagnosed in cattle and experimentally confirmed in mice (Hodge et al., 1988; Fink-Gremmels et al., 1991). Verrucosidin is the most cytotoxic and genotoxic compound among tremorgenic mycotoxins (Núñez et al., 2000; Sabater-Vilar et al., 2003). Based on its cytotoxicity, verrucosidin has been evaluated for its effect against cancer cells (Park et al., 2007; Thomas et al., 2013), making this compound interesting for medical purposes. Despite the importance of this metabolite for human health and its potential benefit in medicine, only few studies have been conducted to elucidate its biosynthesis. Núñez et al. (2000) determined favorable growth conditions of *P. polonicum* for verrucosidin production, whereas Aranda et al. (2002) screened verrucosidin-producer strains to develop a molecular probe to identify mycotoxigenic fungi in foodstuff, which was further used by Rodríguez et al. (2012) to design a TaqMan probe. Nowadays, the elucidated biosyntheses of the structurally related α-pyrone polyenes citreoviridin, aurovertins, and aspernidgulenes (Lin et al., 2016, 2019; Li et al., 2018), available fungal genome sequences (Weber and Kim, 2016; Nielsen and Nielsen, 2017), and bioinformatic tools open up the possibility to identify the biosynthetic genes responsible for verrucosidin biosynthesis.

Biosynthetic gene clusters (BGCs) (Brakhage, 2013; Keller, 2019) consist of central biosynthetic genes, often PKS or/and non-ribosomal peptide synthetase (NRPSs) genes, and are associated with additional genes encoding so-called tailoring enzymes, which are required for the modification of the carbon structure or encode transporters, transcription factors, and resistance proteins. Thousands of gene clusters have been described in fungal genomes, a number that is higher compared to the chemically identified compounds (Yu et al., 2015). Most of these uncharacterized BGCs are often “silent,” meaning their genes are not transcribed under laboratory conditions and a number of strategies exist for their activation (Bergmann et al., 2007, 2010; Fischer et al., 2016). On the other hand, gene deletion on active BGCs can be used to characterize the function of the tailoring genes and to assign a BGC to a compound (Valente et al., 2020).

The clustered regularly interspaced short palindrome repeats (CRISPR) technology is by now the method of choice in genome studies of filamentous fungi (Nødvig et al., 2015; Fang and Tyler, 2016; Pohl et al., 2016; Deng et al., 2017; Krappmann, 2017; Nagy et al., 2017; Nielsen et al., 2017; Weber et al., 2017; Song et al., 2019; Tong et al., 2019; Wang and Coleman, 2019). It allows marker-free genome editing by generating mutations at specific sites in the genome. CRISPR is based on the endonuclease Cas9, that, when associated with small sequences of crRNA (CRISPR-RNA) and tracrRNA (Trans-activating crRNA), usually referred to as guide RNA (gRNA), recognizes genomic protospacer sequences and site-specifically cuts the double strand of DNA (Tong et al., 2019).

In the present manuscript, we described the bioinformatic identification of a number of α-pyrone polyketide synthases (PKSs) in *P. polonicum*. One BGC was selected based on the putative function of the biosynthetic genes of the cluster, and the verrucosidin-encoding PKS was confirmed to be expressed in verrucosidin-producing *Penicillium* strains. Besides, we established the CRISPR-Cas9 technology in *P. polonicum* X6, which helped us characterize the verrucosidin BGC in *P. polonicum*.

## Materials and Methods

### Fungal Strains

*Penicillium polonicum* strain X6 and *P. crustosum* strain CAL64 were isolated from chestnut production chain (Prencipe et al., 2018). *P*enicillium *aurantiogriseum* CBS 112021 was obtained from the Westerdijk Fungal Biodiversity Institute and used as a positive control of verrucosidin production. The strains were grown on Potato Dextrose Agar plates (PDA, Merck KGaA, Darmstadt, Germany) with 50 μg/ml streptomycin (Merck KGaA) in the dark at 25°C for 7–10 days. *P. polonicum* mutants obtained were grown on PDA supplemented with 100 μg/ml of hygromycin B (Thermo Fischer Scientific, Waltham, MA, United States) under the same conditions. All the strains were maintained in glycerol stock at –80°C.

### Growth of Penicillium spp. *in vitro*

Conidial suspensions were obtained by adding 5 ml of sterile water with 0.01% (v/v) Tween-20 and gently scraping the surface of fungal cultures grown on Petri dishes, as in Spadaro et al. (2013). The final conidia concentration was measured using a hemocytometer and adjusted by dilution to different concentrations depending on each assay.

To evaluate verrucosidin production *in vitro*, 1 ml of conidial suspension (10^8^ conidia/ml) was inoculated in 30 ml of malt extract broth (MEB, w/v: 2% malt extract, 2% glucose, 0.1% peptone) and in 30 ml of Czapek yeast broth (CYB, w/v: 0.5% yeast extract, 3.5% Czapek broth). Flasks were kept at 26°C for 10 days, 55% RH with 12 h of light and 12 h of dark. After 10 days of incubation, the fungal mycelium was separated from the liquid media through a double layer of sterile gauze and half of the mycelium was immediately frozen in liquid nitrogen, stored at –80°C, and used for RNA extraction. The remaining fungal tissue was used for verrucosidin extraction.

### Bioinformatic Analyses

To identify BGCs in the genomes of *P. polonicum* strain IBT4502 (GCA_002072265.1) and *P. polonicum* strain hy4 (GCA_003344595.1), antiSMASH (Weber et al., 2015) was used and only clusters containing a putative PKS similar to both CtvA protein (Q0C9L7.1) and AurA (A0A0M4L8I7.1) were further considered (query coverage ≥50% and *e*-value > e^–5^). All genes found to be highly similar to the putative PKS genes were considered as part of the gene cluster whereas proteins with unknown function encoded by genes found far away from the core gene were omitted. The genes showing 70% query coverage and identity with differentially expressed genes (DEGs) of Kim et al. (2016) were also reported. The proteins in these clusters were additionally blasted against *P. expansum* (ALJY00000000.1) to verify their presence. BLAST using Non-Redundant database (Madden, 2013) and Interproscan (Quevillon et al., 2005) was used to find functional domains and predict a putative function.

### DNA Extraction and PCR

Fungal DNA was extracted from mycelium using E.Z.N.A. ^®^Fungal DNA Mini Kit (Omega Bio-tek, Norcross, GA, United States) with brief modifications. The mycelium was lysed by adding the required amount of lysis buffer and two tungsten beads in a TissueLyser II (Qiagen, Hilden, Germany) at 20.00 Hz speed for 20 min. DNA concentration and purity were checked by a spectrophotometer (Nanodrop 2000, Thermo Scientific, Wilmington, DE, United States).

To amplify the genomic DNA (gDNA) of *Penicillium* spp., Taq DNA Polymerase (Qiagen) was used with PCR mixture containing 1 × PCR buffer, 0.2 mM dNTPs, 0.4 μM of each primer, 0.5 U of polymerase, and 10 ng of gDNA. PCR parameters to amplify fragments between 100 and 400 bp were as follows: 3 min at 95°C; 35 cycles of 30 s at 95°C, 30 s at 58°C and 30 s at 72°C; 5 min at 72°C. PCR parameters to amplify fragments between 2 and 3 kbp were as follows: 5 min at 95°C; 35 cycles of 30 s at 95°C, 45 s at 65°C and 3 min at 72°C; 5 min at 72°C.

To amplify DNA repair template from plasmid pUChph1 (Liebmann et al., 2004), the Phusion Flash High-Fidelity PCR Master Mix (Thermo Fischer Scientific) was used according to the manufacturer’s instructions. PCR parameters were as follows: 10 s at 98°C; 30 cycles of 10 s at 98°C, 30 s at 60°C and 20 s at 72°C; 1 min at 72°C. The amplified PCR product was loaded onto an agarose gel, cut out, and extracted using Zymoclean Gel DNA Recovery Kit (Zymo Research, Irvine, CA, United States), according to the manufacturer’s instruction. All primer sequences used in the PCR are listed in Supplementary Table 1.

### RNA Extraction and RT-qPCRs

RNA was extracted from 100 mg of fungal mycelium using Spectrum Plant Total RNA (Sigma-Aldrich, St. Louis, MO, United States), following the manufacturer’s instructions. Mycelium was placed in a 2-ml tube with two tungsten beads and tubes were immersed in liquid nitrogen for 1 min. Then, samples were immediately lysed using TissueLyser II at 20.00 Hz for 1 min.

DNase treatment and first-strand cDNA synthesis were performed according to Valente et al. (2020) using a TURBO DNA-free^TM^ Kit (Thermo Fischer Scientific) and a High Capacity cDNA Reverse Transcription Kit (Thermo Fischer Scientific).

RT-qPCR was performed with StepOne^TM^ and StepOnePlus^TM^ Real-Time PCR System with Power SYBR^TM^ Green PCR Master Mix (Thermo Fischer Scientific); cycling conditions were 5 min at 95°C, followed by 45 cycles of 10 s at 95°C, 30 s at 58°C, and 30 s at 72°C. In order to determine relative gene expression (RGE), the 2^ΔΔcq^ method (Pfaffl, 2001) was used with cDNA of samples, by comparing the amplification of β-tubulin gene with the amplification of the target gene. *P. aurantiogriseum* was used as reference strain for verrucosidin production, as it is reported to be a verrucosidin producer (Fink-Gremmels et al., 1991). All primer sequences used in RT-qPCR reactions are listed in Supplementary Table 1.

### Verrucosidin Extraction

Verrucosidin produced *in vitro* was extracted from approximately 0.5 g of fungal mycelium. The mycelium was placed in 2-ml tubes, and 1.5 ml of MeOH:chloroform (1:2, v/v) was added to the samples, which were then subjected to ultrasound for 30 min. After centrifugation at 4452 × *g* for 5 min, the liquid phase was transferred into a new tube. The mycelium pellet was subjected again to two further extractions: the first with ethyl acetate and the second with isopropanol. The extracts were combined and concentrated at 45°C (Eppendorf concentrator 5301, Hamburg, Germany). The dry extracts were then resuspended in 500 μl of H_2_O:acetonitrile (1:1, v/v) and transferred into a HPLC vial for HPLC-MS/MS analysis.

Verrucosidin produced on apples was extracted from 5 g of decayed tissue around the inoculation site, samples were homogenized with 5 ml of water, and the protocol described in Valente et al. (2020) was adopted to extract verrucosidin from clear juice.

### Chemical Analyses

The HPLC-MS/MS system consisted of a binary pump and a vacuum degasser (1260 Agilent Technologies, Santa Clara, CA, United States) connected to a Varian auto-sampler Model 410 Prostar (Varian, Palo Alto, CA, United States), equipped with a 20-μl loop and coupled with a Varian 310-MS TQ Mass Spectrometer.

To characterize the metabolic profile of wild-type and knockout strains, 10 μl of each extract was analyzed using a C18 analytical column (Luna 3 μm, 150 × 2 mm, 100 Å, Phenomenex, Torrance, CA, United States) and the chromatographic separation was achieved by gradient conditions for 45 min at a flow rate of 300 μl/min. Solvent A was water and solvent B was acetonitrile both containing 0.1% formic acid. The gradient was programmed as follows: 0–3 min isocratic 5% B, followed by a linear gradient to 100% B, ending at 40 min and from 40 to 45 min isocratic 100% B. Full-scan mass spectra were acquired in the positive-ion mode over the *m*/*z* range from 100 to 700 using the TQ mass analyzer.

To perform the verrucosidin qualitative analysis, the same HPLC-MS/MS system was used. HPLC was equipped with a Pursuit XRs ULTRA 2.8 μm C18 (100 × 2 mm, Varian) column and a binary mixture as a mobile phase: solvents A and B were composed of 40 of 0.05% formic acid and 60% of acetonitrile, respectively. The isocratic mode was used at a flow rate of 0.2 L/min for 5 min. A mass spectrometer was equipped with an electrospray ionization (ESI) source operating in positive ion mode, whereas Product Ion Scan (PS) mode was used for triple-quadrupole: *m*/*z* 417→100–427. The collision gas (Ar) pressure was set at 2 mbar.

### Protoplasts Preparation and CRISPR-Cas9 Procedure

Protocols used to obtain protoplasts from *Aspergillus fumigatus* (Weidner et al., 1998), *Ophiostoma piceae* (Wang et al., 1999), *P*enicillium *nalgiovense* (Fierro et al., 2004), *Penicillium paxilli* (Young et al., 1998; McMillan et al., 2003), *Penicillium chrysogenum* (Pohl et al., 2016), *Penicillium crustosum*, and *Penicillium janthinellium* (Nicholson et al., 2015) were used as the basis to design a protocol for protoplast preparation of *P. polonicum*. Fungal mycelium was obtained by inoculating 2 ml of conidial suspension (1.25 × 10^8^ conidia/ml) in 50 ml of YGG (8 g/L KCl, 16 g/L glucose, 6.6 g/L yeast nitrogen base, 1.5 g/L citric acid, 6 g/L KH_2_PO_4_, and 2 g/L yeast extract). Flasks were shaken on a rotary shaker (180 rpm) at 26°C for 20 h. Fungal mycelium was filtered through Miracloth (Merck KGaA) and washed with MgSO_4_ 0.6 M. A pre-treatment was conducted by mixing 5 g of mycelium in 50-ml tubes with 5 mM Na_2_EDTA and 25 mM 2-mercaptoethanol; tubes were kept in horizontal position in a rotary shaker (80 rpm) at 30°C for 20 min. Fungal mycelium was washed with 0.6 M MgSO_4_ and then digested with 2 g of Vinotaste^®^Pro (Lamothe-Abiet, Canéjan, France) and 0.1 g of Lysing Enzymes from *Trichoderma harzianum* (Sigma-Aldrich) suspended in 15 ml of Osmo Solution (1.2 M MgSO_4_, 10 mM sodium phosphate buffer). The lytic solution was kept at 30°C, 80 rpm. Every 30 min, the mycelium-lysis solution was mixed using a serological pipette and visually checked under a microscope; about 3–4 h were necessary to digest the cell wall and obtain protoplasts. The suspension was filtered through Miracloth in order to separate and remove undigested mycelia. Protoplasts were then recovered using Trapping Buffer (0.6 M sorbitol, 0.1 M Tris–HCl, pH 7) and by centrifuging for 25 min. The intermediate layer was separated and washed twice with STC Buffer (2.4 M sorbitol, 10 mM CaCl_2_, and 10 mM Tris–HCl, pH 7.5). All centrifugation steps were performed at 3,000 × *g* at 4°C. Finally, protoplasts were resuspended in 1 ml of STC Buffer and kept on ice until transformation with PEG.

CRISPR RNAs were designed using Alt-R Custom Cas9 crRNA (CRISPR RNA) Design Tool^1^ and are listed in Supplementary Table 1. The off-target analysis was performed using Blast. Alt-R^®^ CRISPR-Cas9 crRNA, Alt-R^®^ CRISPR-Cas9 tracrRNA (trans-activating crRNA), and Alt-R^®^ S.p. Cas9 nuclease V3 were purchased from IDT (Integrated DNA Technologies, Inc., Coralville, IA, United States) and were combined to obtain a ribonucleoprotein (RNP) complex according to the manufacturer’s instructions. Briefly, crRNA (2 nM) and tracrRNA (2 nM) were heated at 95°C for 5 min and cooled at room temperature to obtain gRNA. Three microliters of each gRNA was mixed with 4 μl of Cas9 at room temperature for 20 min. The entire mixture of RNP together with 10 μl of repair template (3 μg) and 40 μl of PEG20 solution (20% PEG6000) were gently mixed with 80 μl of protoplasts (10^7^ protoplasts/ml). As a control, instead of DNA, 10 μl of water was used. The mixtures were kept on ice for 30 min, and then 900 μl of PEG60 solution (60% of PEG6000) was added. After 30 min of incubation on ice, protoplasts were gently spread on Petri dishes with regeneration media (RM; w/v: Malt extract 2%, peptone 1%, glucose 2%; sucrose 0.8 M) supplemented with or without hygromycin.

### Characterization of Mutants *in vitro*

Mutants were selected on PDA supplemented with hygromycin B. Conidia were obtained in order to assure uniformity in the genetic material. The deletion event was confirmed by PCR, sequencing PCR products, and Southern blot. Southern blot analysis was performed according to Stroe et al. (2020), and the probes used are listed in Supplementary Table 1.

Deletion mutants were compared with wild-type *P. polonicum* X6 by inoculating 5 μl of spore suspension (1 × 10^6^ conidia/ml) on PDA and incubating the plates at 25°C in the dark. Colony diameter (cm) and number of asexual spores (conidia/plate) were measured up to 7 days post inoculation (dpi). Additionally, deletion mutants were inoculated in liquid media (CYB and MEB) as previously described in order to examine verrucosidin production.

### Characterization of Mutants *in vivo*

Apples cv. Ambrosia and cv. Opal were surface disinfected and wounded as described in Zhang et al. (2012). Ten microliters of conidial suspension (1 × 10^6^ conidia/ml) of each strain was pipetted into each wound, whereas controls were inoculated with deionized Ringer solution. Inoculated apples were placed in plastic trays, covered with a transparent polyethylene film, and stored for 14 days at room temperature. To verify the pathogenicity of *P. polonicum* X6, apples cv. Gala were used.

### Statistical Analyses and Software

All statistical analyses were performed with one-way ANOVA followed by Tukey’s *b* multiple comparison test using IBM SPSS statistics software version 24 (SPSS Inc., Chicago, IL, United States); *p* < 0.05 was considered significant.

SnapGene software (from Insightful Science; available at snapgene.com) was used to draw gene clusters and visualize annotated DNA sequences.

## Results

### Putative Verrucosidin Gene Cluster

**FIGURE 1:**
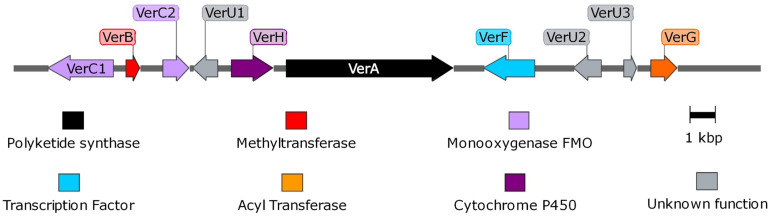
Representation of verrucosidin gene cluster in *P. polonicum*. Maps were obtained with SnapGene software.

**FIGURE 2:**
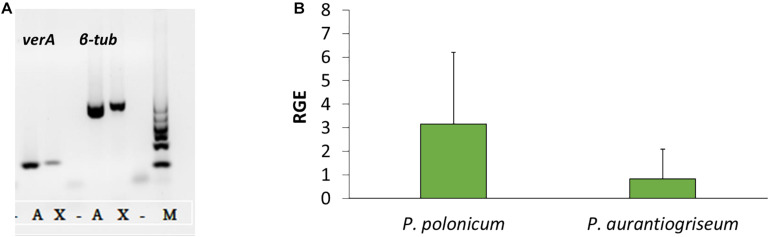
Presence and expression of *verA* gene in *Penicillium* spp. Amplification of *verA* and β-tubulin genes from gDNA **(A)**: X, *P. polonicum* X6; A, *P. aurantiogriseum* CBS121001; M, GelPilot 100 bp Ladder; –, negative control (PCR mix without DNA). Relative gene expression (RGE) of *verA* (*cl4A*) gene in *P. polonicum* and *P. aurantiogriseum* **(B)** was evaluated 10 days post inoculation on CYB. The expression is relative to the expression of the β-tubulin gene, and *P. aurantiogriseum* was used as reference strain.

Using the PKS genes for aurovertin (AurA) and citreoviridin (CtvA) biosyntheses as matrix, 16 putative BGCs (Supplementary Figure 1) were found in the genome of *P. polonicum*. Of them, 10 clusters were present in both available *P. polonicum* genomes, whereas clusters 4 (later identified as verrucosidin BGC), 12, and 13 were only present in *P. polonicum* IBT 4502 and clusters 14, 15, and 16 in *P. polonicum* strain hy4 genome (Supplementary Table 2). Cluster 4 was further investigated due to the presence of genes encoding putative tailoring enzymes involved in verrucosidin biosynthesis (Figure 1): a putative methyltransferase (named *cl4B* or *verB*), two Flavin adenine dinucleotide (FAD)-dependent monooxygenases (*cl4C1* and *cl4C2* or *verC1* and *verC2*), an acyltransferase (*cl4G* or *verG*), and a cytochrome P450 (*cl4H* or *verH*) gene. To monitor verrucosidin production, *P. polonicum* X6, *P. aurantiogriseum* CBS 112021, and *P. crustosum* CAL64 were grown in induction media. As shown in Supplementary Figure 2, *P. polonicum* and *P. aurantiogriseum* were able to produce verrucosidin, whereas *P. crustosum* failed to produce this compound. Consistently, we could verify the corresponding PKS gene sequence in the genomes of *P. polonicum* X6 and *P. aurantiogriseum* CBS 112021 by PCR, but not in the *P. crustosum* genome (Figure 2A). Moreover, the expression of the *verA* gene was confirmed both in *P. polonicum* X6 and in *P. aurantiogriseum* CBS 112021 by qPCR (Figure 2B).

### Deletion of PKS Through CRISPR-Cas9

To investigate the role of *verA* in verrucosidin biosynthesis, the gene was deleted using CRISPR-Cas9. For this purpose, a method to obtain protoplasts for *P. polonicum* strain X6 was developed. The method allowed obtaining 1 × 10^7^ protoplasts/ml starting from 5 g of mycelium.

**FIGURE 3:**
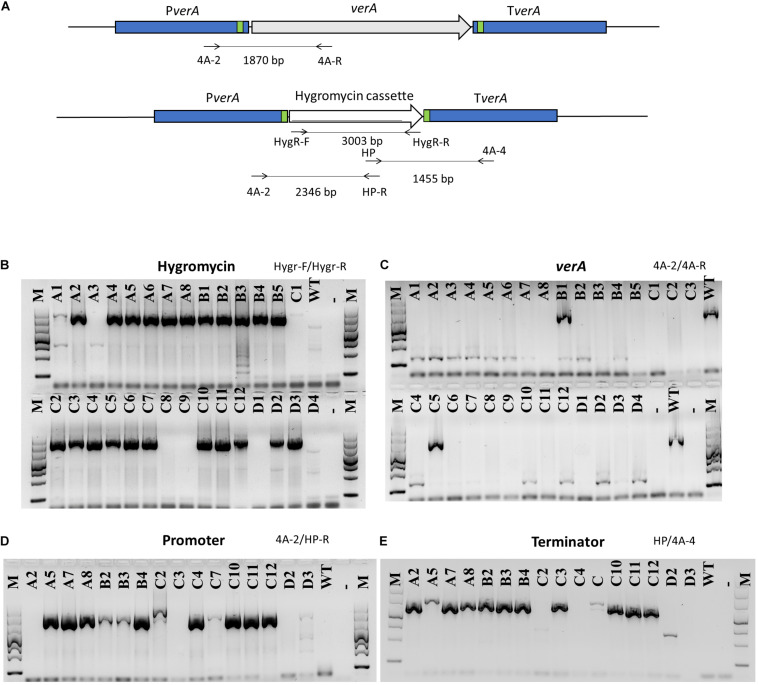
PCR analysis of *verA* mutants. Schematic presentation of the *verA* (*cl4A*) locus in the wild-type and deletion mutants; homology sequences (50 bp) are indicated in green, and primers used are marked by arrows **(A)**. Amplification of hygromycin resistance cassette **(B)** and *verA* gene **(C)**; confirmation of orientation of inserted repair DNA **(D,E)**; M, GelPilot Wide Range Ladder; WT, wild-type *P. polonicum* X6; A–D, deletion mutants for *verA*; –, negative control (PCR mix without DNA).

In order to delete target genes with the endonuclease Cas9, the protospacers were designed based on the sequenced promoter and terminator of target genes in *P. polonicum* X6. To obtain the deletion mutants, two sets of gRNA were used, allowing to cut both the promoter and the terminator and to excise the target gene. The endonuclease Cas9 was mixed *in vitro* with gRNA and a repair DNA template encoding the hygromycin resistance cassette. The repair DNA template was amplified using primers with an additional tail of 50 bp providing at both flanking regions DNA micro-homology close to the PAM site. This way, the correct integration of the repair DNA through homology-mediated end joining (MMEJ) was achieved. Obtained knockout mutants were assessed through PCR, amplifying both the hygromycin resistance cassette and the target gene (Figures 3A–C). Sixteen mutants, in that the hygromycin resistance cassette was successfully amplified whereas amplification of the *verA* PKS gene failed, were randomly chosen and the correct integration of the repair template was confirmed using PCR with primer pairs designed inside the *hph* gene and in the promoter and terminator region of the deleted PKS gene (Figures 3A,D,E). In most cases, correct Δ*verA* mutants were found (Figures 3D,E). Some of the positive transformants were further confirmed by sequencing the amplified PCR products (Supplementary Figure 3) and by Southern blot analysis (Supplementary Figure 4). The results showed that one of the strains displayed a repetition of 50 bp of micro-homology in the promoter region (Supplementary Figure 3), whereas Δcl4A_C10, Δcl4A_C11, and Δcl4A_C12 mutants displayed a correct integration of the *hph* gene and lack of ectopic integrations (Supplementary Figures 3, 4) and were therefore further studied.

**FIGURE 4:**
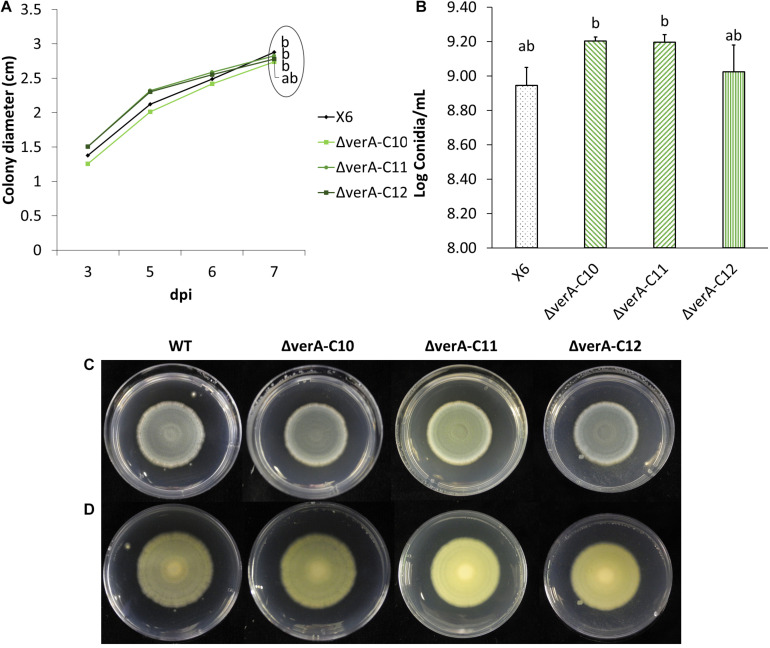
Effect of *verA* deletion on *P. polonicum* growth *in vitro.* Colony diameter after 3 to 7 days post inoculation **(A)** conidia production **(B)** and plate view (front **C** and reverse **D**) at 7 days post inoculation (dpi) on PDA. WT, wild-type *P. polonicum* X6; ΔverA, deletion mutants for *verA* (*cl4A*). Values followed by the same letter are not statistically different by Tukey’s *b* multiple comparison test (*p* < 0.05).

### Phenotype of Mutants

Three *P. polonicum* X6 *verA* mutants were phenotypically characterized *in vitro* and on apples. Compared to wild-type strain, the mutant strains displayed the same ability to grow (Figures 4A,C,D) and no significant differences in conidiation (Figure 4B). Analyzing the metabolic profile of wild-type strain and knockout mutants, the verrucosidin chromatographic peak was absent in deletion mutants (Supplementary Figure 5), independently of the used induction medium (Table 1 and Supplementary Figure 6). By contrast, verrucosidin formation in *P. polonicum* X6 was not influenced, when the PKS gene of another α-pyrone BGC, *cl3A*, was deleted (data not shown).

**TABLE 1:** Effect of gene deletion on verrucosidin production *in vitro* and *in vivo.*

**Strain**	**CYB**	**MEB**	**APPLE**
*P. aurantiogriseum* CBS 112021*	+	+	+
*P. polonicum* X6 wild-type	+	+	+
*P. polonicum* X6 Δcl4A_C10	−	−	−
*P. polonicum* X6 Δcl4A_C11	−	−	−
*P. polonicum* X6 Δcl4A_C12	−	−	−

Penicillium polonicum wild type, *P. polonicum* deletion mutants, and *P. aurantiogriseum* were inoculated in CYB and MEB broth or on apples. Presence (+) or absence (−) of verrucosidin production at 10 days post inoculation.

*Penicillium aurantiogriseum CBS 112021 was used as a qualitative standard since the verrucosidin standard is not commercially available.

**FIGURE 5:**
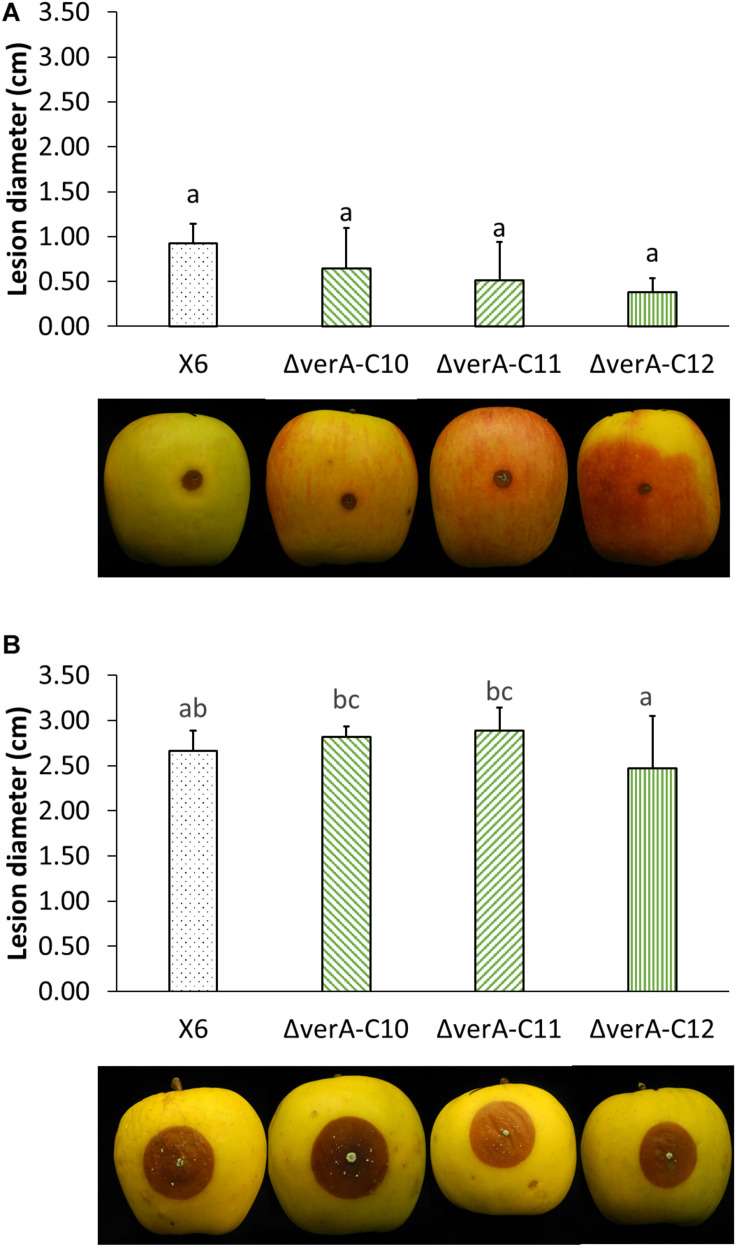
Analysis of *verA* deletion on the virulence of *P. polonicum in vivo.* Lesion diameter (cm) and pictures of apples cv. Ambrosia **(A)** and Opal **(B)** were recorded 14 days after inoculation. WT, wild-type *P. polonicum* X6; ΔverA, deletion mutants for *verA* (*cl4A*). Values followed by the same letter are not statistically different by Tukey’s *b* multiple comparison test (*p* < 0.05).

The pathogenicity of the wild-type strain X6 of *P. polonicum* was previously verified on apple (Supplementary Table 3). The effect of gene deletion on the virulence of *P. polonicum* was assessed on apple, as it is a host species for this pathogen and the virulence of the fungal strain can be quantified by measuring the lesion diameter, whereas on chestnut, the symptoms are mild and not easily measured. The virulence was tested on two apple cultivars. On apples cv. Ambrosia, *verA* deletion mutants displayed a slightly reduced virulence 7 days after the inoculation (Supplementary Table 4), whereas this behavior was not observed after 14 days of storage (Figure 5A) or on apples cv. Opal (Figure 5B).

## Discussion

We report the identification of the BGC for the α-pyrone polyketide verrucosidin in the genome of *P. polonicum*, a known verrucosidin producer (Frisvad et al., 2004). Initial similarity analyses with the PKS genes for aurovertin (AurA) and citreoviridin (CtvA) had ended up with 16 BGCs putatively encoding the biosynthesis of α-pyrone polyketides. Besides *P. polonicum*, we confirmed *P. aurantiogriseum* to be able to produce verrucosidin (Fink-Gremmels et al., 1991), whereas *P. crustosum* did not produce this compound, as expected (Frisvad et al., 2004). Under the used laboratory conditions, meaning cultivation in different induction media, the *verA* gene was expressed and verrucosidin formation was verified in both producer species *P. polonicum* X6 and *P. aurantiogriseum* CBS 112021.

To delete *verA*, encoding a putative highly reducing polyketide synthase (HR-PKS), for the first time an *in vitro* method for CRISPR-Cas9 gene deletion was established in *P. polonicum* X6. Up to the present, CRISPR-Cas9 technology was adopted only in the *Penicillium* species *P. chrysogenum*. Protoplasts of a strain harboring the Cas9 gene on an AMA1 plasmid were transformed with a synthesized gRNA (Pohl et al., 2016). In this work, the method of Al Abdallah et al. (2017) was followed, who successfully transformed protoplasts of *A. fumigatus* with RNP and a repair template harboring antibiotic resistance to select mutants. This strategy does not require the generation of plasmids or the expression of the endonuclease Cas9, but is based on transforming protoplasts with the endonuclease protein Cas9 together with crRNA and tracrRNA. Therefore, there is an immediate cleavage activity when the RNP is targeted into the cells and the RNP is degraded by the host cell within a short period, reducing the possibility of off-target activity (Wang and Coleman, 2019). This strategy requires both accurate design of the crRNA and a protocol to transform fungi. A method to obtain protoplasts in *P. polonicum* X6 was optimized based on available protocols used for other filamentous fungi (Weidner et al., 1998; Young et al., 1998; Wang et al., 1999; McMillan et al., 2003; Fierro et al., 2004; Nicholson et al., 2015; Pohl et al., 2016) and allows obtaining a high amount of protoplasts. Mutants for putative PKS gene were obtained, confirming that the CRISPR-Cas9 method is effective and could be used for gene editing in *P. polonicum*. The finding of a small number of mutants displaying incorrect insertion of the foreign DNA necessitate screening and confirmation of mutants by molecular techniques such as PCR and Southern blot analysis. As an example, when sequencing the terminator region of the deleted *verA* gene in the mutant B4, we observed an unexpected insertion, that suggested correct cutting of the double-strand DNA by the endonuclease, but missing MMEJ. To our knowledge, there were no previous reports about MMEJ occurring only at one excision site. Therefore, the integration of repair DNA could have occurred by non-homologous end-joining (Krappmann, 2017). The efficiency of homologous recombination could be improved by adjusting the length of the homology sequence or by changing the amount of donor DNA, as previously shown in *A. fumigatus* (Al Abdallah et al., 2017).

Deletion mutants for the HR-PKS encoded by *verA* were no longer able to produce verrucosidin *in vitro* confirming that *verA* is responsible for the biosynthesis of this compound. The here identified gene cluster was bioinformatically hypothesized as verrucosidin cluster in *P. polonicum* by Li et al. (2018), but this prediction was not experimentally proven. Surprisingly, the verrucosidin gene cluster was absent in the genome of *P. polonicum* strain hy4, confirming high variability in secondary metabolism between strains of the same species (Ballester et al., 2015). The biosynthetic genes were also absent in *P. expansum* strain NRRL 62431. This fungal genome was initially deposited as *P. aurantiogriseum* and later assigned to another species (Ballester et al., 2015). Due to the lack of verrucosidin gene cluster, we can conclude that both *P. expansum* strains NRRL 62431 and *P. polonicum* strain hy4 are not able to produce this SM.

Secondary metabolites are known to play a central role in many biological processes such as growth development (Calvo et al., 2002) and pathogenesis (Scharf et al., 2014; Macheleidt et al., 2016), which were extensively reviewed (Fox and Howlett, 2008; Keller, 2019). In this work, the deletion of the *verA* gene did not affect *P. polonicum* in terms of growth rate and conidiation on PDA. Concerning the effect of deletion on the virulence of the strains, apples were used as preferential host, as *P. polonicum* is reported as a postharvest pathogen on pome fruit (Scholtz and Korsten, 2016; Ouhibi et al., 2018). The pathogenicity of the wild-type strain X6 of *P. polonicum* was previously verified on apple, where the virulence is easily quantified by measuring the lesion diameter. Slight differences in virulence of *ΔverA* mutants were observed on apple cv. Ambrosia, whereas this behavior was not observed on apples cv. Opal. These results are not surprising, as the apple cultivar is known to be a key factor in the virulence, such as in the case of patulin biosynthesis, which may be considered a cultivar-dependent aggressiveness factor (Banani et al., 2016; Snini et al., 2016). However, a reduced virulence of *ΔverA* mutants was observed only during the first steps of blue mold development on apple fruit; therefore, further studies are needed to confirm these preliminary observations.

## Conclusion

The verrucosidin biosynthesis BGC was identified by bioinformatic and genetic approaches. Sixteen HR-PKS gene-containing BGCs were discovered in the available genomes of *P. polonicum*. These BGCs are probably involved in the biosynthesis of α-pyrone type polyketides, which could have many biological activities, such as cytotoxic, antitumor, antimicrobial, and anti-germination activities and are therefore a potential source of novel compounds (Schäberle, 2016; Li et al., 2018; Stroe et al., 2020). Based on the putative function of the genes present in the clusters, one of these BGCs was further characterized through deletion, in order to investigate its involvement in verrucosidin biosynthesis. To delete the selected PKS gene, CRISPR-Cas9 was adopted in *P. polonicum*. This allowed obtaining mutants by replacing the target genes with repair template sequences with 50 bp homology to chromosomal sequences. By applying this technology, the verrucosidin BGC was confirmed by creating deletion mutants for *verA* gene coding for HR-PKS known to be the key enzyme of the biosynthesis. The availability of the CRISPR-Cas technology will allow characterizing the biosynthetic potential of this interesting fungal species.

## Data Availability Statement

The datasets presented in this study can be found in online repositories. The names of the repository/repositories and accession number(s) can be found in the article/Supplementary Material.

## Author Contributions

SV, EP, VS, TH, AB, and DS conceived and designed the study. SV performed the experiments. EP, VS, and GM performed bioinformatic, Southern blot, and chemical analyses, respectively. SV and EP retrieved the literature and drafted the manuscript. All authors reviewed and approved the final manuscript.

## Conflict of Interest

The authors declare that the research was conducted in the absence of any commercial or financial relationships that could be construed as a potential conflict of interest.

## Footnotes

1 https://eu.idtdna.com/site/order/designtool/index/CRISPR_CUSTOM
