# Supplementary material for: CRISPR-Cas9-Based Discovery of the Verrucosidin Biosynthesis Gene Cluster in Penicillium polonicum
Source: Front Microbiol. 2021 May 21;12:660871. doi: 10.3389/fmicb.2021.660871 (PMC8176439; doi:10.3389/fmicb.2021.660871)
Supplement: Supplementary file 1 [file Image_1.pdf]

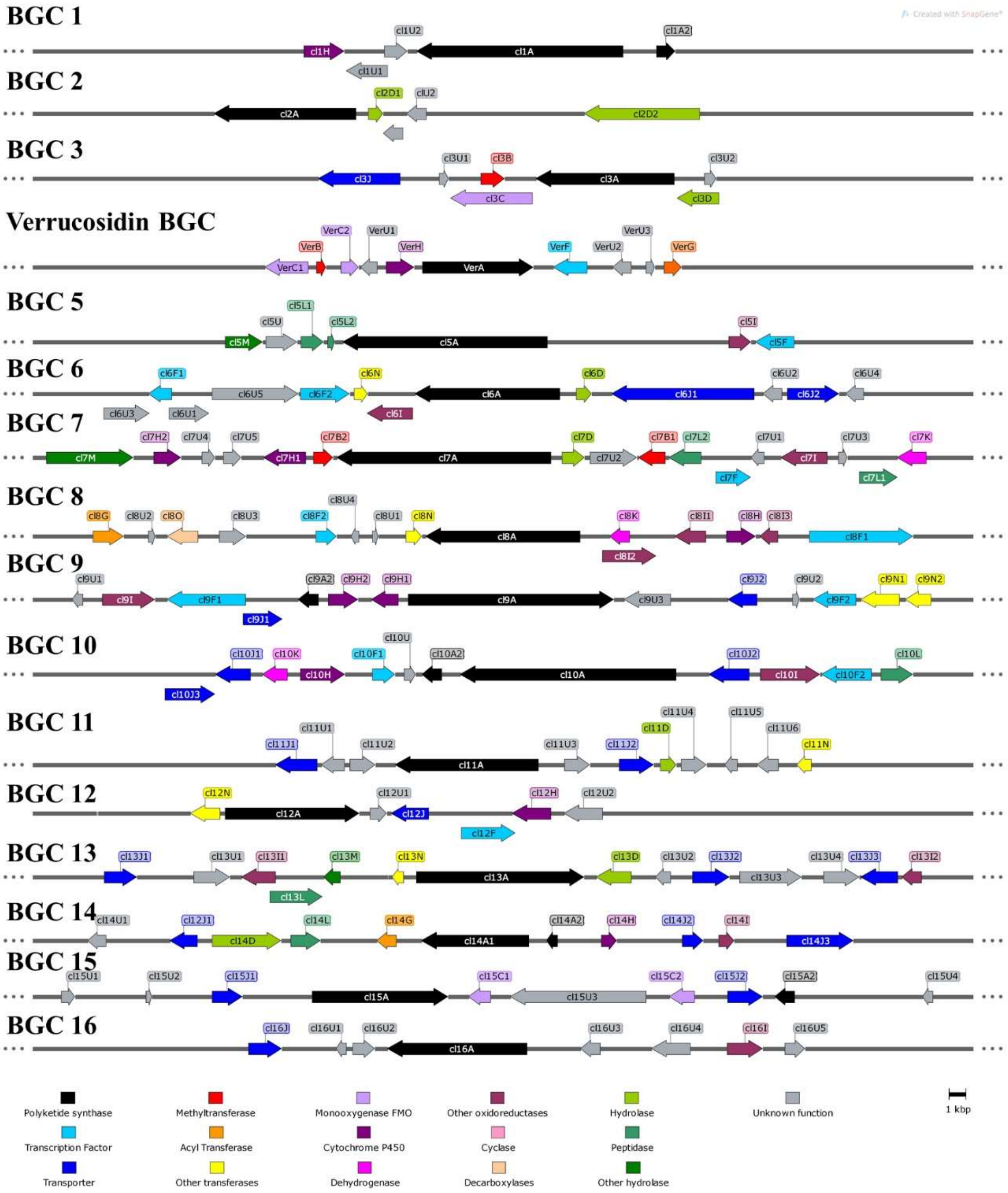


**Supplementary Figure 1.** Representation of 16 HR-PKS containing BGCs found in *P. polonicum* genomes. Genes are marked by arrows and putative function is given by colours. The fourth cluster was later confirmed to be the verrucosidin cluster. Maps were obtained with Snapgene software.
