# Supplementary material for: CRISPR-Cas9-Based Discovery of the Verrucosidin Biosynthesis Gene Cluster in Penicillium polonicum
Source: Front Microbiol. 2021 May 21;12:660871. doi: 10.3389/fmicb.2021.660871 (PMC8176439; doi:10.3389/fmicb.2021.660871)
Supplement: Supplementary file 2 [file Image_2.pdf]

A

Strain	MEB	CYB
P. polonicum X6	+	+
P. aurantiogriseum CBS 112021	+	+
P. crustosum CAL64	-	-

B

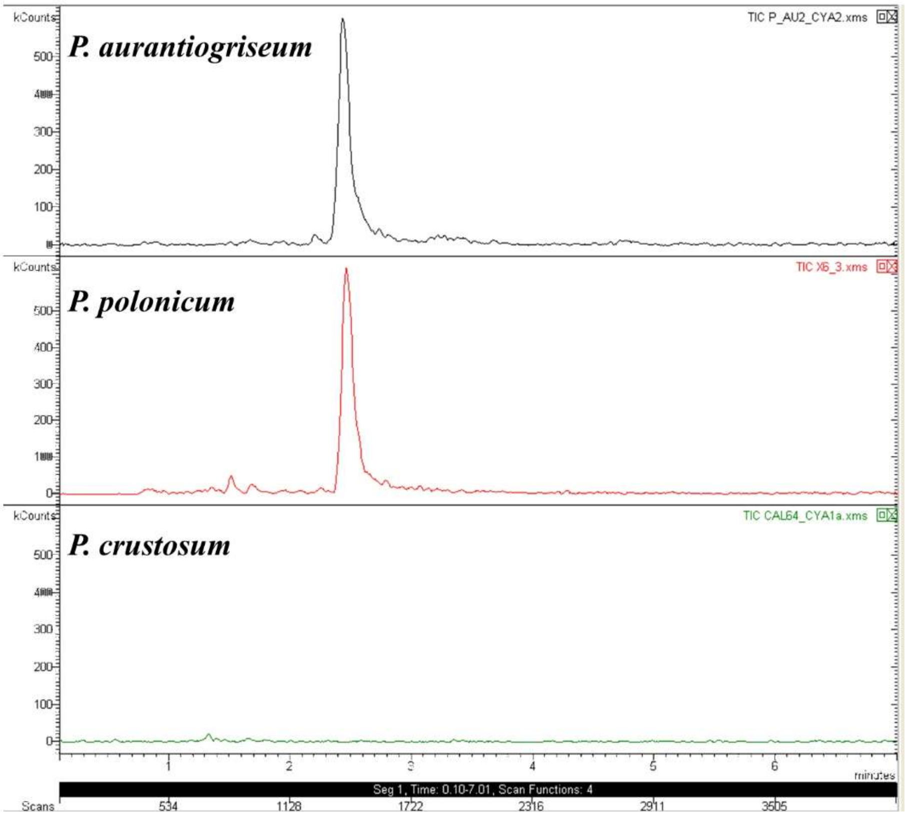

Supplementary Figure 2. Verrucosidin production *in vitro*. *P. polonicum, P. aurantiogriseum* and *P. crustosum* were inoculated in CYB and MEB broth. Presence (+) or absence (-) of verrucosidin production 10 days after inoculation (A) and chromatograms on CYB (B).
