# Supplementary material for: CRISPR-Cas9-Based Discovery of the Verrucosidin Biosynthesis Gene Cluster in Penicillium polonicum
Source: Front Microbiol. 2021 May 21;12:660871. doi: 10.3389/fmicb.2021.660871 (PMC8176439; doi:10.3389/fmicb.2021.660871)
Supplement: Supplementary file 3 [file Image_3.pdf]

Promoter *verA*

WT GCATCGGAGGAGGGTATTCAAGCTTGT**CTACCAACATCATTGATAGAAGTACAAAATTACATTCCTTATCATAG**CCTACCGTCCACTGGTTGAGTACATGATTGCAAGGTTG

ΔA7 GCATCGGAGGAGGGTATTCAAGCTTGT**CTACCAACATCATTGATAGAAGTACAAAATTACATTCCTTATCATAG**CCTAGCTTGCATGCCTGCAGGTCGACTCTAGATGCAT

ΔA8 GCATCGGAGGAGGGTATTCAAGCTTGT**CTACCAACATCATTGATAGAAGTACAAAATTACATTCCTTATCATAG**CCTAGCTTGCATGCCTGCAGGTCGACTCTAGATGCAT

ΔB4 GCATCGGAGGAGGGTATTCAAGCTTGT**CTACCAACATCATTGATAGAAGTACAAAATTACATTCCTTATCATAG**CCTAGCTTGCATGCCTGCAGGTCGACTCTAGATGCAT

ΔC10 GCATCGGAGGAGGGTATTCAAGCTTGT**CTACCAACATCATTGATAGAAGTACAAAATTACATTCCTTATCATAG**CCTAGCTTGCATGCCTGCAGGTCGACTCTAGATGCAT

ΔC11 GCATCGGAGGAGGGTATTCAAGCTTGT**CTACCAACATCATTGATAGAAGTACAAAATTACATTCCTTATCATAG**CCTAGCTTGCATGCCTGCAGGTCGACTCTAGATGCAT

ΔC12 GCATCGGAGGAGGGTATTCAAGCTTGT**CTACCAACATCATTGATAGAAGTACAAAATTACATTCCTTATCATAG**CCTAGCTTGCATGCCTGCAGGTCGACTCTAGATGCAT

Terminator *verA*

WT TTGCTGACGTGATTTGCTTTCGACTATCCG**GTGGATCCCTTGGCGTGTCAGCAACTTGCATCGTATGCCCAGGTGCCCAG**GTGTTCGATTTGAAAATCCGGTTGCCTTCCCAA

ΔA7 CACGCGAAGGGCGAATTCCAGCACACTG**GTGGATCCCTTGGCGTGTCAGCAACTTGCATCGTATGCCCAGGTGCCCAG**GTGTTCGATTTGAAAATCCGGTTGCCTTCCCAA

ΔA8 CACGCGAAGGGCGAATTCCAGCACACTG**GTGGATCCCTTGGCGTGTCAGCAACTTGCATCGTATGCCCAGGTGCCCAG**GTGTTCGATTTGAAAATCCGGTTGCCTTCCCAA

ΔB4 CACGCGAAGGGCGAATTCCAGCACACTG**GTGGATCCCTTGGCGTGTCAGCAACTTGCATCGTATGCCCAGGTGCCCAG**GATCCCTTGGCGTGTCA**GCAACTTGCATCGTAT GCCCAGGTGCCCAG**GTGTTCGATTTGAAAATCCGGTTGCCTTCCCAA

ΔC10 CACGCGAAGGGCGAATTCCAGCACACTG**GTGGATCCCTTGGCGTGTCAGCAACTTGCATCGTATGCCCAGGTGCCCAG**GTGTTCGATTTGAAAATCCGGTTGCCTTCCCAA

ΔC11 CACGCGAAGGGCGAATTCCAGCACACTG**GTGGATCCCTTGGCGTGTCAGCAACTTGCATCGTATGCCCAGGTGCCCAG**GTGTTCGATTTGAAAATCCGGTTGCCTTCCCAA

ΔC12 CACGCGAAGGGCGAATTCCAGCACACTG**GTGGATCCCTTGGCGTGTCAGCAACTTGCATCGTATGCCCAGGTGCCCAG**GTGTTCGATTTGAAAATCCGGTTGCCTTCCCAA

Supplementary Figure 3. Sequencing of deletion locus. Blue = protospacer, red = PAM, green = hygromycin cassette, in bold = 50 bp homology, underline = expected site of cut, WT = *P. polonicum* X6, Δ = deletion mutants for *verA* (*cl4A*) gene.
