# Supplementary material for: CRISPR-Cas9-Based Discovery of the Verrucosidin Biosynthesis Gene Cluster in Penicillium polonicum
Source: Front Microbiol. 2021 May 21;12:660871. doi: 10.3389/fmicb.2021.660871 (PMC8176439; doi:10.3389/fmicb.2021.660871)
Supplement: Supplementary file 4 [file Image_4.pdf]

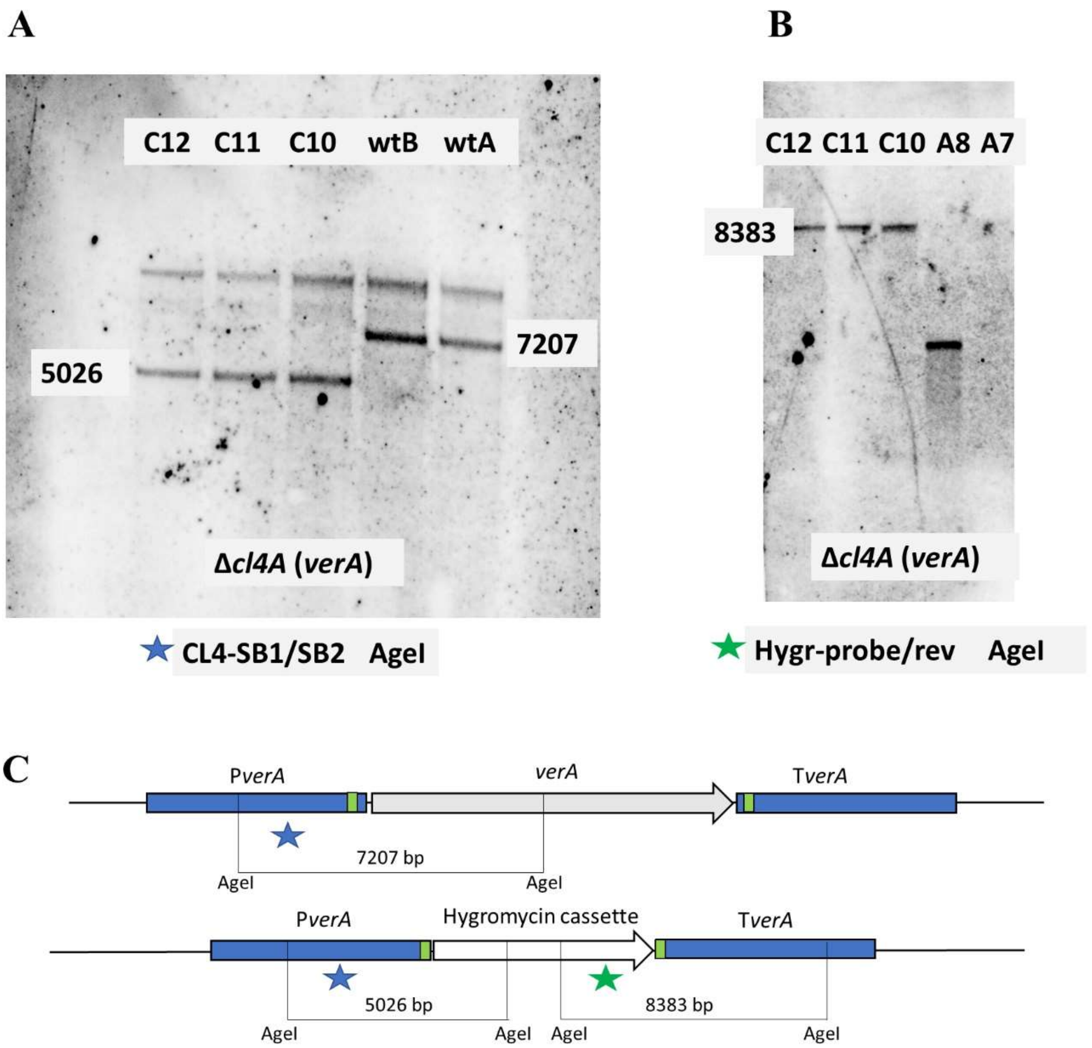

Supplementary Figure 4. Southern blot verification of correct integration of transgene (A) and absence of ectopic integrations in the genome (B), and schematic presentation of the *verA* (*cl4A*) locus in the wild-type and deletion mutants (C). DNA of wild-type *P. polonicum* and Δ*cl4A* (*verA*) mutants was digested with AgeI restriction endonuclease; the probes are marked as a coloured star.
