# Supplementary material for: CRISPR-Cas9-Based Discovery of the Verrucosidin Biosynthesis Gene Cluster in Penicillium polonicum
Source: Front Microbiol. 2021 May 21;12:660871. doi: 10.3389/fmicb.2021.660871 (PMC8176439; doi:10.3389/fmicb.2021.660871)
Supplement: Supplementary file 5 [file Image_5.pdf]

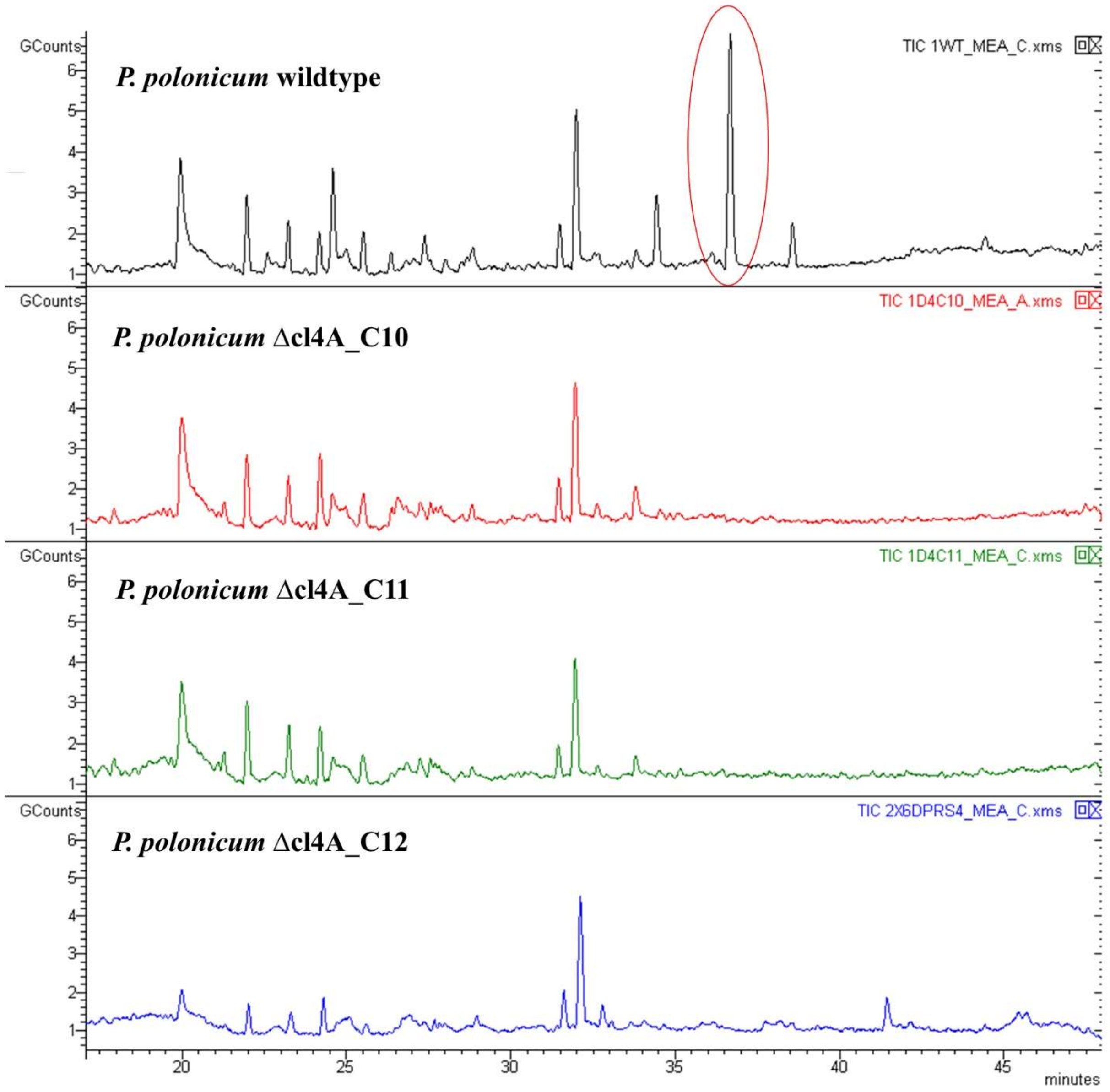

Supplementary Figure 5. Metabolic profile of wild type and knockout strains. Liquid chromatography-mass spectrometry (LC-MS) spectrum of wild-type, Δcl4A_C10, Δcl4A_C11 and Δcl4A_C12 extracts. Verrucosidin chromatographic peak is encircled in red.
