# Supplementary material for: CRISPR-Cas9-Based Discovery of the Verrucosidin Biosynthesis Gene Cluster in Penicillium polonicum
Source: Front Microbiol. 2021 May 21;12:660871. doi: 10.3389/fmicb.2021.660871 (PMC8176439; doi:10.3389/fmicb.2021.660871)
Supplement: Supplementary file 6 [file Image_6.pdf]

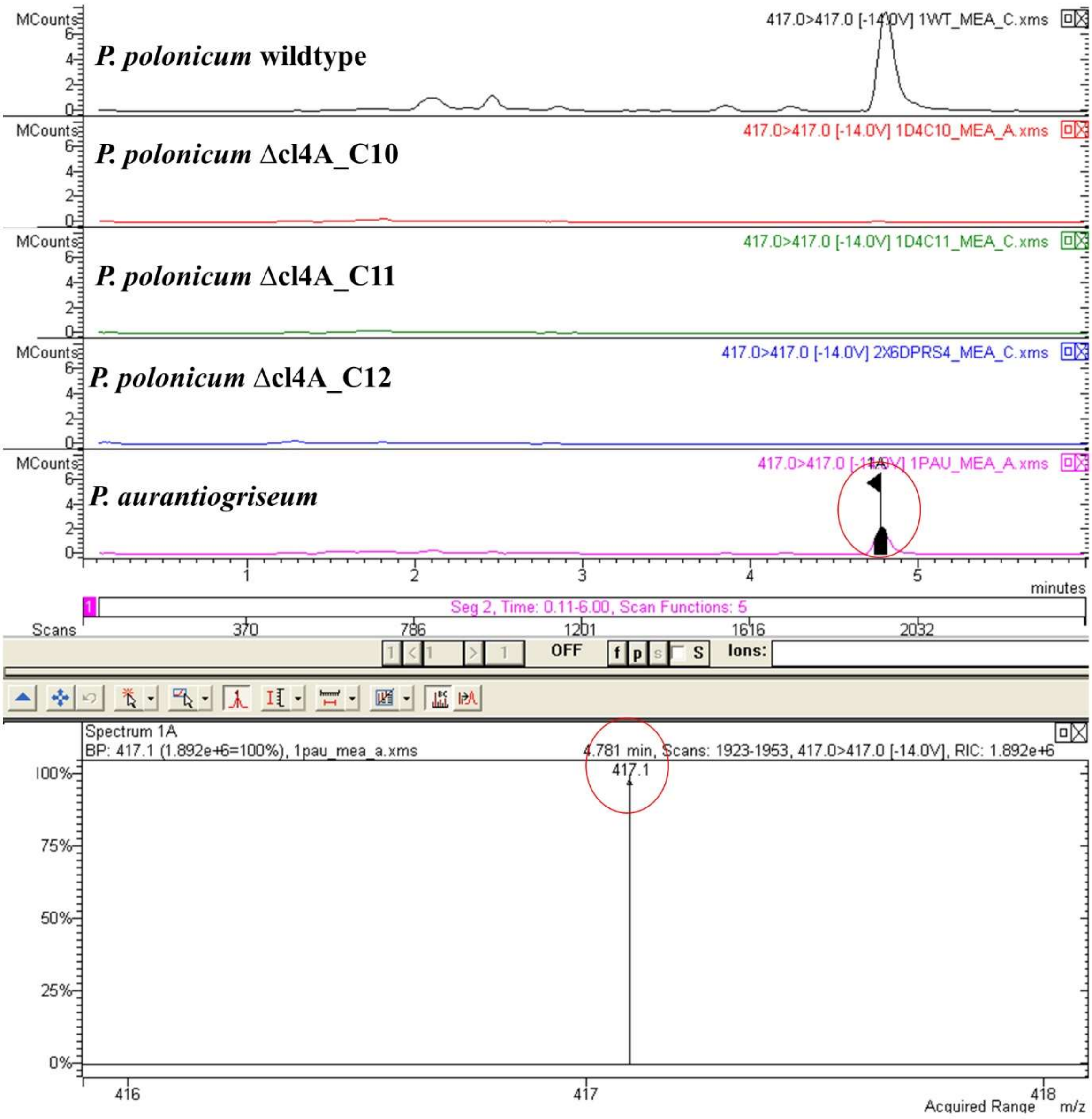

Supplementary Figure 6. Effect of *verA* deletion on *P. polonicum* verrucosidin production *in vitro.* Liquid chromatography-mass spectrometry (LC-MS) spectrum of *P. polonicum* wild-type, Δcl4A mutants and *P. aurantiogriseum* extracts. Verrucosidin chromatographic peak is encircled in red. Mass spectrum related to the chromatographic peak circled in red, which confirms the identity of verrucosidin (MW = 417). *P. aurantiogriseum* CBS 112021 able to produce verrucosidin was used as qualitative standard since the verrucosidin standard is not commercially available.
