# Supplementary material for: CRISPR-Cas9-Based Discovery of the Verrucosidin Biosynthesis Gene Cluster in Penicillium polonicum
Source: Front Microbiol. 2021 May 21;12:660871. doi: 10.3389/fmicb.2021.660871 (PMC8176439; doi:10.3389/fmicb.2021.660871)
Supplement: Supplementary file 7 [file Table_1.pdf]

Supplementary Table 1. Primers and sequences used in this study

RT-qPCR and PCR with gDNA to verify presence of genes		
Name	Sequences	Amplification
4A-F	CCTCTATGTTGGGTCGATCAAG	*verA*/ *cl4A*
4A-R	ATAGCAGGTTTGGGAAGATGG	
BTUB-F	CGAGTTGACCCAGCAGATGT	β-tubulin
BTUB-R	GTCTGGACGTTGTTGGGGAT	
**Primers to amplify donor DNA (hygromycin resistance cassette)**		
Name	Sequence	Amplification
H50P4-F	CTACCAACATCATTGATAGAAGTACAAAATTACATTCC TTATCATAGCCTAGCTTGCATGCCTGCAGGTC	50 bp micro-homology with *verA* promoter and terminator in bold
H50P4-R	CTGGGCACCTGGGCATACGATGCAAGTTGCTGACACG CCAAGGGATCCACCAGTGTGCTGGAATTCGCCCTTC	
**Primers to verify knockout mutants**		
Name	Sequence	Amplification
Hygr-R	CAGTGTGCTGGAATTCGCCCTTC	Hygromycin resistance cassette
Hygr-F	AGCTTGCATGCCTGCAGGTC	
4A-2	TGTGCAGTTGGCTTTCATTTC	Promoter of *verA*(*cl4A*)
HP	CCGCAAGGAATCGGTCAATACA	
4A-4	GATGACCTCTTGGCGCTTAT	Terminator of *verA*(*cl4A*)
HP-R	TGTATTGACCGATTCCTTGCGG	
CL4-SB1	CTGACAGACCGGCCAATAAAAG	Southern blot probe
CL4-SB2	CAAGAAGATCGACCAGCATACC	
Hygr-probe	CCGCAAGGAATCGGTCAATACA	Southern blot probe on hygromycin resistance cassette
Hygr-rev	CAGTGTGCTGGAATTCGCCCTTC	
**Protospacer and Protospacer Adjacent Motif (PAM)**		
Name	Sequence – PAM	On target score
CD.Cas9.TP PB7515.AE	GTACTCAACCAGTGGACGGT-AGG	80
CD.Cas9.FW KX5170.AA	TGACACGCCAAGGGATCCAC-CGG	91
