# Supplementary material for: CRISPR-Cas9-Based Discovery of the Verrucosidin Biosynthesis Gene Cluster in Penicillium polonicum
Source: Front Microbiol. 2021 May 21;12:660871. doi: 10.3389/fmicb.2021.660871 (PMC8176439; doi:10.3389/fmicb.2021.660871)
Supplement: Supplementary file 8 [file Table_2.pdf]

Supplementary Table 2. Sixteen BGCs found in the genome of *P. polonicum* strains IBT 4502 and/or hy4 are reported. Gene names are chosen according to previously identified genes in aurovertin biosynthesis:, cl = cluster, ver = verrucosidin BGC, A = polyketide synthase, B = methyltransferase, C = Monooxygenase FMO, D = Hydrolase, E = Cyclase, F = Transcription Factor, G = Acyl Transferase, H = Cytochrome P450, I = Other oxidoreductases, J = Transporter, K = Dehydrogenase, L = Peptidase, M = Other hydrolase, N = Other transferases, O = decarboxylases, U = Unknown function. PKS in bold has similarity with *ctvA* and *aurA*. The name of protein in *P. polonicum* and presence (+) or absence (-) of the protein in the genome of other *Penicillium* spp. are reported. In putative function is reported the closest match considering BLAST and INTERPROSCAN. In notes, additional domains or genes overexpressed or underexpressed *in P. expansum* while producing verrucosidin are reported (Kim et al., 2016).

Name	*P. polonicum* IBT 4502	*P. polonicum* hy4	*P. expansum* NRRL 62431	Putative function	Notes
cl1H	OQD68095.1	+	+	Cytochrome P450 oxydoreductase	
cl1U1	OQD68222.1	+	+	Hypothetical protein	
cl1U2	OQD67563.1	+	+	Calycin	
cl1A	OQD67773.1	+	+	Polyketide synthase	
cl1A2	OQD68121.1	+	+	Polyketide synthase, enoylreductase, alchool deydrogenase	

Name	*P. polonicum* IBT 4502	*P. polonicum* hy4	*P. expansum* NRRL 62431	Putative function	Notes
cl2A	OQD63672.1	+	+	Polyketide synthase	
cl2D1	OQD63761.1	+	+	Hypotetical protein with serin hydrolase domain	
cl2U1	OQD63370.1	+	+	Hypothetical protein	Overexpressed in verrucosidin producing conditions
cl2U2	OQD63792.1	+	+	Endosomal protein with GOLD domain (protein-protein interaction) and transmembrane domains	
cl2U3	-	-	-	Hypothetical protein	
cl2D2	OQD63654.1	+	+	Hypothetical protein with glycoside hydrolase domain	

Name	*P. polonicum* IBT 4502	*P. polonicum* hy4	*P. expansum* NRRL 62431	Putative function	Notes
*cl3*J	OQD60362.1	+	-	ABC transporter	
*cl3U*1	OQD60333.1	+	-	Protein of unknown function DUF3716, Phospolipase A2, active site	
*cl3*C	OQD60328.1	+	+	FAD-dependent pyridine nucleotide-disulphide oxidoreductase	
*cl3*B	OQD60332.1	+	+	S-adenosyl-L-methionine-dependent methyltransferase	Overexpressed in verrucosidin producing conditions
*cl3*A	OQD60358.1	+	+	Polyketide synthase	Additional Enoyl reductase domain
*cl3*D	OQD60365.1	+	+	Serine hydrolase FSH	
*cl3U*2	OQD60340.1	+	-	Hypothetical protein	

Name	*P. polonicum* IBT 4502	*P. polonicum* hy4	*P. expansum* NRRL 62431	Putative function	Notes
cl4C1/ verC1	OQD69144.1	-	-	FAD/NAD(P)-binding domain-containing protein	Similar to CTVC
cl4B/ verB	OQD68888.1	-	-	Methyltransferase	Similar to CTVB
cl4C2/ verC2	OQD69870.1	-	-	Monooxygenase FAD-binding protein	Similar to CTVC
cl4U1/ verU1	OQD69720.1	-	-	Hypothetical protein with transmembrane domains	
cl4H/ verH	OQD69071.1	-	-	Cytochrome P450	
cl4A/ verA	OQD69647.1	-	-	Polyketide synthase	
cl4F/ verF	OQD68940.1	+	+	Transcription factor	Underexpressed in verrucosidin producing conditions
cl4U2/ verU2	OQD69889.1	+	+	Hypothetical protein	
cl4U3/ verU3	OQD69759.1	+	+	Hypothetical protein	
cl4G/ verG	OQD69357.1	+	+	Acyl-CoA N-acyltransferase	

Name	*P. polonicum* IBT 4502	*P. polonicum* hy4	*P. expansum* NRRL 62431	Putative function	Notes
*cl5*A	OQD63426.1	+	-	Polyketyde synthase	
*cl5L*1	OQD63428.1	+	-	Peptidase C45, acyl-coenzyme A:6-aminopenicillanic acid acyl-transferase	
cl5I	OQD63430.1	+	+	Cytochrome b561	
cl5L2	OQD63436.1	+	-	Peptidase C45, acyl-coenzyme A:6-aminopenicillanic acid acyl-transferase	
cl5U	OQD63551.1	+	+	Hypothetical protein	
*cl5*F	OQD63610.1	+	-	Transcription factor	
cl5M	OQD63635.1	+	+	Alpha-L-fucosidase	

Name	*P. polonicum* IBT 4502	*P. polonicum* hy4	*P. expansum* NRRL 62431	Putative function	Notes
cl6J1	OQD61059.1	+	+	Cation transporting ATPase	
cl6U1	OQD61070.1	+	+	Translation elongation factor	
cl6A	OQD61128.1	+	-	Polyketide synthase	Additional Enoyl reductase domain
cl6U2	OQD61131.1	+	+	Eukaryotic translation initiation factor 2	
cl6D	OQD61132.1	+	+	Serine hydrolase FSH	
cl6J2	OQD61154.1	+	+	Sulfate transporter	
cl6U3	OQD61177.1	+	+	Hypotetical protein	
cl6U4	OQD61186.1	+	+	CENP-A-nucleosome distal centromere subunit CENP-L	
cl6I	OQD61187.1	+	+	Aldo/keto reductase	
cl6N	OQD61204.1	+	+	Hypotetical protein with Protein kinase-like domain	
cl6F1	OQD61216.1	+	+	DNA-binding HORMA	
cl6F2	OQD61240.1	+	+	Fungal transcriptional regulatory protein	Overexpressed in verrucosidin producing conditions
cl6U5	OQD61244.1	+	+	DNA-directed RNA polymerase	

Name	*P. polonicum* IBT 4502	*P. polonicum* hy4	*P. expansum* NRRL 62431	Putative function	Notes
cl7U1	OQD64350.1	+	-	Hypothetical protein with EthD domain	
cl7B1	OQD64367.1	+	-	O-methyltransferase	
cl7D	OQD64429.1	+	-	Alpha/beta hydrolase	
cl7L1	OQD64470.1	+	+	Peptidase M24	
cl7L2	OQD64500.1	+	-	Peptidase M24	
cl7U2	OQD64508.1	+	-	Acetate-CoA ligase	
cl7I	OQD64517.1	+	-	Monooxygenase	
cl7F	OQD64519.1	+	-	C6 finger transcription factor	
cl7H1	OQD64603.1	+	-	Cytochrome P450	
cl7K	OQD64659.1	+	-	SirQ protein	
cl7U3	OQD64682.1	+	-	Hypothetical protein	
cl7B2	OQD64738.1	+	-	Methyltransferase SirN-like protein	
cl7U4	OQD64754.1	-	-	Hypothetical protein	
cl7M	OQD64757.1	+	+	Protein phosphatase, Leucine rich repeats	
cl7A	OQD64783.1	+	-	Polyketide synthase	
cl7U5	OQD64845.1	+	-	Translation elongation factor eEF 1	
cl7H2	OQD64894.1	+	+	Cytochrome P450	

Name	*P. polonicum* IBT 4502	*P. polonicum* hy4	*P. expansum* NRRL 62431	Putative function	Notes
cl8A	OQD61269.1	+	-	Polyketide synthase	
cl8F1	OQD61286.1	+	+	Transcription factor jumonji	
cl8N	OQD61288.1	+	-	Aminoglycoside phosphotransferase	
cl8I1	OQD61304.1	+	-	FAD-dependent isoamyl alcohol oxidase	
cl8G	OQD61318.1	+	+	Acyl-CoA N-acyltransferase	
cl8U1	OQD61322.1	+	+	Hypothetical protein	
cl8I2	OQD61330.1	+	-	Oxidoreductase, NAD(P)-binding protein	
cl8K	OQD61332.1	+	-	Zinc-binding dehydrogenase	
cl8H	OQD61338.1	+	-	Cytochrome P450	
cl8I3	OQD61339.1	+	-	Oxidoreductase, NAD(P)-binding protein	
cl8U2	OQD61365.1	+	-	Hypothetical protein	
cl8O	OQD61370.1	+	+	Oxalyl-CoA decarboxylase	
cl8U3	OQD61375.1	+	+	Hypothetical protein	
cl8F2	OQD61382.1	+	-	Fungal transcriptional regulatory protein	
cl8U4	OQD61435.1	-	-	Hypothetical protein	

Name	*P. polonicum* IBT 4502	*P. polonicum* hy4	*P. expansum* NRRL 62431	Putative function	Notes
*cl9*H1	OQD62930.1	+	-	Cytochrome P450	
*cl9U*1	OQD62937.1	+	-	Hypothetical protein	
*cl9*J1	OQD62943.1	+	-	MFS general substrate transporter	
cl9N1	OQD62951.1	+	-	Hypothetical protein with protein kinase-like domain	
*cl9*J2	OQD62954.1	-	+	Amino acid transporter, transmembrane	
cl9N2	OQD62956.1	+	-	Calcium/calmodulin-dependent protein kinase II isoform	
cl9I	OQD62974.1	+	-	NAD(P)-binding protein	
*cl9*A2	OQD63049.1	+	-	GroES-like protein, enoylreductase	
*cl9*H2	OQD63117.1	+	-	Cytochrome P450	
cl9U2	OQD63150.1	+	-	Hypothetical protein	
*cl9*F1	OQD63214.1	+	+	Hypothetical potrein with Zn2C6 DNA binding domain	
*cl9*F2	OQD63223.1	+	-	Fungal transcriptional regulatory protein	
cl9U3	OQD63280.1	+	-	Hypothetical protein	
*cl9*A	OQD63284.1	+	-	Polyketide synthase	

Name	*P. polonicum* IBT 4502	*P. polonicum* hy4	*P. expansum* NRRL 62431	Putative function	Notes
cl10I	OQD60326.1	+	+	Taurine catabolism dioxygenase TauD/TfdA	
*cl10*A2	OQD60329.1	+	+	Polyketide synthase, enoylreductase, alchool deydrogenase	
cl10U	OQD60337.1	+	+	Hypotetical protein	
cl10K	OQD60359.1	+	+	acyl-CoA dehydrogenase	
*cl10*J1	OQD60366.1	+	+	Amino acid/polyamine transporter I	
*cl10*J2	OQD60369.1	+	+	Sucrose/H+ symporter	
*cl10*F1	OQD60370.1	+	+	Fungal transcriptional regulatory protein	
*cl10*H	OQD60373.1	+	+	Cytochrome P450	
*cl10*F2	OQD60384.1	+	+	Transcription factor	
*cl10*J3	OQD60393.1	+	+	Sucrose/H+ symporter	Underexpressed in verrucosidin producing conditions
*cl10*A	OQD60396.1	+	-	Polyketide synthase	
cl10L	OQD60403.1	+	+	Peptidase M24	

Name	*P. polonicum* IBT 4502	*P. polonicum* hy4	*P. expansum* NRRL 62431	Putative function	Notes
cl11J1	OQD65718.1	+	+	Transport protein	
cl11U1	OQD65662.1	-	+	Hypothetical protein	
cl11U2	OQD65737.1	+	+	Cyclin-like	
cl11A	OQD66219.1	+	-	Polyketyde synthase	
cl11U3	OQD65636.1	+	-	Polysaccharide synthase	
cl11J2	OQD66164.1	-	-	MFS transporter	
cl11D	OQD65818.1	-	+	alpha/beta-hydrolase	
cl11U4	OQD65688.1	+	+	Autophagy-related protein 3	
cl11U5	OQD65906.1	+	+	Hypothetical protein	
cl11U6	OQD66060.1	-	+	Hypothetical protein	
cl11N	OQD65617.1	-	+	Hypothetical protein/Glycosyl transferase	

Name	*P. polonicum* IBT 4502	*P. polonicum* hy4	*P. expansum* NRRL 62431	Putative function	Notes
cl12N	OQD59992.1	-	-	Putative aminotransferase	
cl12A	OQD59991.1	-	-	Polyketyde synthase	
cl12U1	OQD59995.1	-	-	putative toxin biosynthesis protein	
cl12J	OQD59999.1	-	+ (vaguely similar)	MFS transporter	
cl12F	OQD59997.1	-	-	Fungal specific transcription factor	
cl12H	OQD59998.1	-	-	Cytochrome P450	
cl12U2	OQD59994.1	-	-	NAD dependent epimerase/dehydratase	

Name	*P. polonicum* IBT 4502	*P. polonicum* hy4	*P. expansum NRRL 62431*	Putative function	Notes
cl13J1	OQD61546.1	-	-	Putative transporter	
cl13U1	OQD61463.1	-	-	Hypothetical protein	
cl13I1	OQD61510.1	-	+	Fatty acid hydroxylase	
cl13L	OQD61571.1	-	-	Amidase family protein	
cl13M	OQD61479.1	-	+	Acyl-CoA thioesterase	
cl13N	OQD61620.1	-	-	3-amino-3-carboxypropyl transferase	
cl13A	OQD61552.1	-	-	Polyketyde synthase	
cl13D	OQD61622.1	-	+	Hydrolase	
cl13U2	OQD61573.1	-	-	Hypothetical protein DUF3632	
cl13J2	OQD61591.1	-	+	MFS transporter	
cl13U3	OQD61653.1	-	+	Hypothetical protein with NACHT domain	
cl13U4	OQD61560.1	-	-	Hypothetical protein DUF3176	
cl13J3	OQD61616.1	-	-	MFS transporter	
cl13I2	OQD61608.1	-	-	Hypothetical protein/NADP-binding	

Name	*P. polonicum* IBT 4502	*P. polonicum* hy4	*P. expansum* NRRL 62431	Putative function	Notes
cl14U1	+	QPIC01000001_1_region004_polonicum_gene196	+	Allantoicase	
cl12J1	+	QPIC01000001_1_region004_polonicum_gene197	+	Major facilitator superfamily, general substrate transporter	
cl14D	-	QPIC01000001_1_region004_polonicum_gene198	+	Glycoside hydrolase	
cl14L	-	QPIC01000001_1_region004_polonicum_gene199	+	Peptidase C45, acyl-coenzyme A:6-aminopenicillanic acid acyl-transferase	
cl14G	+	QPIC01000001_1_region004_polonicum_gene200	'	CoA-dependent acyltransferases	
cl14A1	-	QPIC01000001_1_region004_polonicum_gene201	-	Polyketyde synthase	
cl14A2	+	QPIC01000001_1_region004_polonicum_gene202	+	Polyketide synthase, enoylreductase	
cl14H	+	QPIC01000001_1_region004_polonicum_gene203	+	cytochrome P450	
cl14J2	-	QPIC01000001_1_region004_polonicum_gene204	+	Major facilitator superfamily, general substrate transporter	
cl14I	+	QPIC01000001_1_region004_polonicum_gene205	+	Cytochrome b561/ferric reductase transmembrane	
cl14J3	-	QPIC01000001_1_region004_polonicum_gene206	-	ABC transporter	

Name	*P. polonicum* IBT 4502	*P. polonicum* hy4	*P. expansum* NRRL 62431	Putative function	Notes
cl15U1	-	QPIC01000002_1_region002 _polonicum_gene37	-	Hypothetical protein	
cl15U2	+	QPIC01000002_1_region002 _polonicum_gene38	+	Hypothetical protein DUF1993	
cl15J1	+	QPIC01000002_1_region002 _polonicum_gene39	+	Amino acid/polyamine transporter I	Overexpressed in verrucosidin producing conditions
cl15A	-	QPIC01000002_1_region002 _polonicum_gene40	-	Polyketyde synthase	
cl15C1	-	QPIC01000002_1_region002 _polonicum_gene41	-	Hypothetical protein/FAD-linked oxidoreductase	
cl15U3	-	QPIC01000002_1_region002 _polonicum_gene42	-	Hypothetical protein	
cl15C2	-	QPIC01000002_1_region002 _polonicum_gene43	-	Hypothetical protein/FAD-dependent monooxygenase	
cl15J2	-	QPIC01000002_1_region002 _polonicum_gene44	-	Sucrose/H+ symporter	
cl15A2	-	QPIC01000002_1_region002 _polonicum_gene45	-	Polyketide synthase, enoylreductase, alchool deydrogenase	
cl15U4	-	QPIC01000002_1_region002 _polonicum_gene46	-	Hypothetical protein	

Name	*P. polonicum* IBT 4502	*P. polonicum* hy4	*P. expansum* NRRL 62431	Putative function	Notes
cl16J	+	QPIC01000004_1_region001_polonicum_gene191	+	Putative transporter	
cl16U1	+	QPIC01000004_1_region001_polonicum_gene192	+	Hypothetical protein/ribosomal	
cl16U2	+	QPIC01000004_1_region001_polonicum_gene193	+	Hypothetical protein	
cl16A	-	QPIC01000004_1_region001_polonicum_gene194	+	Polyketide synthase	Additional ER domain
cl16U3	+	QPIC01000004_1_region001_polonicum_gene195	+	Hypotetical protein/Heat shock protein	
cl16U4	+	QPIC01000004_1_region001_polonicum_gene196	+	Hypothetical protein/Lipid binding	
cl16I	+	QPIC01000004_1_region001_polonicum_gene197	+	Malic oxidoreductase (NADP-dependent)	
cl16U5	+	QPIC01000004_1_region001_polonicum_gene198	+	Aldolase-type TIM barrel	
