# Supplementary material for: CRISPR-Cas9-Based Discovery of the Verrucosidin Biosynthesis Gene Cluster in Penicillium polonicum
Source: Front Microbiol. 2021 May 21;12:660871. doi: 10.3389/fmicb.2021.660871 (PMC8176439; doi:10.3389/fmicb.2021.660871)
Supplement: Supplementary file 9 [file Table_3.pdf]

Supplementary Table 3. Pathogenicity of *P. polonicum* X6 on apples cv. Gala. It is reported lesion diameter (cm) with standard deviation measured 7, 10 and 14 days after inoculation on apples cv. Gala. Statistical analysis was performed using Student's t-test at 95% and 99% confidence level to compare treated and control fruits.

strain	Day 7		Day 10		Day 14	
P. polonicum	2.06 ± 0.55	$P<0.01$	2.90 ±0.73	$P<0.01$	4.10 ± 1.04	$P<0.01$
Control	0		0		0	
