# Supplementary material for: CRISPR-Cas9-Based Discovery of the Verrucosidin Biosynthesis Gene Cluster in Penicillium polonicum
Source: Front Microbiol. 2021 May 21;12:660871. doi: 10.3389/fmicb.2021.660871 (PMC8176439; doi:10.3389/fmicb.2021.660871)
Supplement: Supplementary file 10 [file Table_4.pdf]

Supplementary Table 4. Effect of *verA* deletion on virulence the of strains of *P. polonicum in vivo*. The mean lesion diameter (cm) with standard deviation measured 7, 10 and 14 days after inoculation on apples cv. Ambrosia and Opal is reported. WT = wild-type *P. polonicum* X6, ΔverA = deletion mutants for *verA* (*cl4A*). Values followed by the same letter are not statistically different by Tukey's-b multiple comparison test ($p < 0.05$).

	cv. Opal				cv. Ambrosia			
strain	7		10		7		10	
WT	0.64 ± 0.42	bc	0.76 ±0.19	cd	1.63 ± 0.13	abc	2,12 ± 0,16	b
ΔverA-C10	0.29 ± 0.15	a	0.47 ± 0.27	abc	1.64 ± 0.16	abc	2,16 ± 0,12	b
ΔverA-C11	0.27 ± 0.15	a	0.40 ± 0.21	ab	1.72 ± 0.11	bc	2,23 ± 0,09	b
ΔverA-C12	0.18 ± 0.12	a	0.27 ± 0.13	a	1.51 ± 0.27	a	1,83 ± 0,46	a
